# The genome sequence of the thistle gall fly, *Urophora cardui *(Linnaeus 1758)

**DOI:** 10.12688/wellcomeopenres.22919.1

**Published:** 2024-09-03

**Authors:** Leila Franzen, Liam M. Crowley, Nathan Medd, University of Oxford and Wytham Woods Genome Acquisition Lab, Darwin Tree of Life Barcoding collective, Wellcome Sanger Institute Tree of Life Management, Samples and Laboratory team, Wellcome Sanger Institute Scientific Operations: Sequencing Operations, Wellcome Sanger Institute Tree of Life Core Informatics team, Tree of Life Core Informatics collective, Darwin Tree of Life Consortium

**Affiliations:** 1 University of Bath, Bath, England, UK; 2 University of Oxford, Oxford, England, UK; 3 University of Edinburgh, Edinburgh, Scotland, UK

**Keywords:** Urophora cardui, thistle gall fly, genome sequence, chromosomal, Diptera

## Abstract

We present a genome assembly from an individual female thistle gall fly, *Urophora cardui* (Arthropoda; Insecta; Diptera; Tephritidae). The genome sequence has a total length of 837.80 megabases. Most of the assembly is scaffolded into 6 chromosomal pseudomolecules. The mitochondrial genome has also been assembled and is 20.37 kilobases in length.

## Species taxonomy

Eukaryota; Opisthokonta; Metazoa; Eumetazoa; Bilateria; Protostomia; Ecdysozoa; Panarthropoda; Arthropoda; Mandibulata; Pancrustacea; Hexapoda; Insecta; Dicondylia; Pterygota; Neoptera; Endopterygota; Diptera; Brachycera; Muscomorpha; Eremoneura; Cyclorrhapha; Schizophora; Acalyptratae; Tephritoidea; Tephritidae; Tephritinae; Myopitini; *Urophora*; *Urophora cardui* (Linnaeus 1758) (NCBI:txid503482).

## Background

*Urophora cardui* (Linnaeus 1758), commonly known as the Canada Thistle Gall Fly or Thistle Stem Gall Fly, is a fruit fly in the family Tephritidae. The adult fly possesses a gloss black thorax, matte black abdomen, light cream to white scutellum, and a cream head dusted in rusty orange. Its wings are clear with distinct dark bands that, unlike others in the genus, fuse at the hind margin of the wing to form distinct a ‘M’ shape ( White, 1988).

*Urophora cardui* occurs throughout temperate Central Europe, with records spanning from the United Kingdom in the west to Southern Russia and the shores of Lake Baikal in the east. It has also been introduced to North America as a biological control agent to manage the population of its primary host plant, the Canada or Creeping Thistle ( *Cirsium arvense*), an invasive weed in that region ( Peschken & Harris, 1975). Its distribution is more latitudinally constrained with most records coming from between 45° and 65° North in its native Palearctic range between 35° and 50° North in its introduced Nearctic range ( GBIF.org, 2024). *U. cardui* is able to disperse relatively large distances ( Eber & Brandl, 1997; Schlumprecht, 1989) and the northern extent of its range may be increasing due to warmer summers, as demonstrated by observations in Finland ( Jansson, 1991).

*Urophora cardui* is a stem-gall-forming parasite of *Cirsium* thistles. In the central European and western parts of its range it is restricted to *C. arvense*. However, at the extremes of its range it switches host: to *C. creticum* in the eastern Mediterranean region ( White & Korneyev, 1989) and to *C. setosum* in the east, between Ukraine and northern Kazakhstan ( Korneyev & White, 1996). In both cases this host shift seems to reflect the relative rarity of the fly’s preferred host in these regions. In the wild females oviposit around 130 to 150 eggs in their lifetime divided into small clutches of between 1 and 12 offspring per multilocular gall ( Freese & Zwölfer, 1996). Eggs, laid in the vegetative shots, have an incubation period of 6.3 days at 24 °C ( Peschken & Harris, 1975). Galls develop around 15 days after oviposition and begin maturation at approximately 36 days, at which stage the larva enters 3rd instar and gains weight rapidly ( Lalonde & Shorthouse, 1985). At between 60 to 100 days post-oviposition, galls contain mature larvae which diapause overwinter and pupate within the gall during spring ( Peschken & Harris, 1975). Adults emerge in early summer with most UK records falling between June and September ( NBN Atlas Partnership, 2024).

Various aspects of this species ecology and life history have been studied due to its potential use as a classical biological control agent in North America ( Peschken & Derby, 1997; Peschken & Harris, 1975; Zwölfer *et al.*, 1970). Since its first release in British Columbia in 1974 subsequent releases in Canada and the USA have established some seemingly stable populations which are thought to partially contribute to the ongoing control of *C. arvense* through stunting and reduced seed production ( De Clerck-Floate & Cárcamo, 2011; Peschken *et al.*, 1982; Winston *et al.*, 2016).

More recently this species, and its hymenopteran parasites, have been studied for a range of different topics: gall evolution ( Zwölfer, 1979), biogeographic dynamics ( Eber & Brandl, 1996), host-parasitoid interactions ( Johannesen & Seitz, 2003; Zwölfer *et al.*, 2007; Zwölfer & Arnold-Rinehart, 1994), and population genetics ( Eber & Brandl, 1997; Johannesen *et al.*, 2010; Seitz & Komma, 1984; Steinmetz *et al.*, 2004). It is hoped that the high-quality reference genome presented here will be of great value to all these fields, especially the latter.

## Genome sequence report

**Figure 1.**
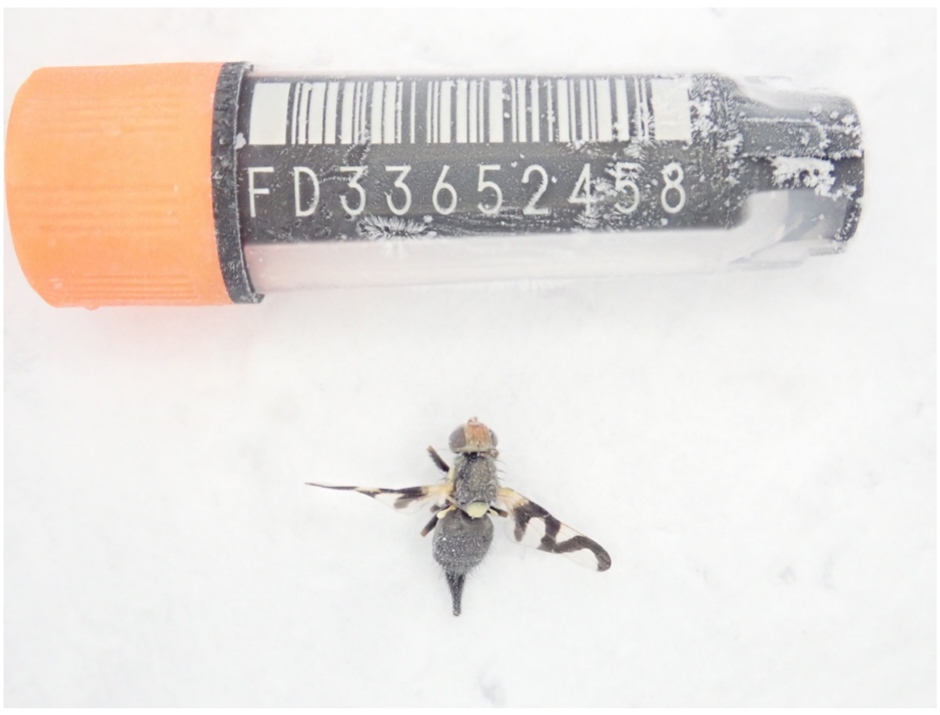
Photograph of the *Urophora cardui* (idUroCard1) specimen used for genome sequencing.

The genome of an adult female *Urophora cardui* ( Figure 1) was sequenced using Pacific Biosciences single-molecule HiFi long reads, generating a total of 27.86 Gb (gigabases) from 2.05 million reads, providing approximately 31-fold coverage. Primary assembly contigs were scaffolded with chromosome conformation Hi-C data, which produced 134.28 Gbp from 889.27 million reads, yielding an approximate coverage of 160-fold. Specimen and sequencing information is summarised in Table 1.

**Table 1.** Specimen and sequencing data for *Urophora cardui*.

Project information			
**Study title**	Urophora cardui (thistle gall fly)		
**Umbrella BioProject**	PRJEB62614		
**Species**	*Urophora cardui*		
**BioSample**	SAMEA112232592		
**NCBI taxonomy ID**	503482		
Specimen information			
**Technology**	**ToLID**	**BioSample accession**	**Organism part**
**PacBio long read sequencing**	idUroCard1	SAMEA112233058	Whole organism
**Hi-C sequencing**	idUroCard1	SAMEA112233058	Whole organism
Sequencing information			
**Platform**	**Run accession**	**Read count**	**Base count (Gb)**
**Hi-C Illumina NovaSeq 6000**	ERR11496088	8.89e+08	134.28
**PacBio Sequel IIe**	ERR11483519	2.05e+06	27.86

**Figure 2.**
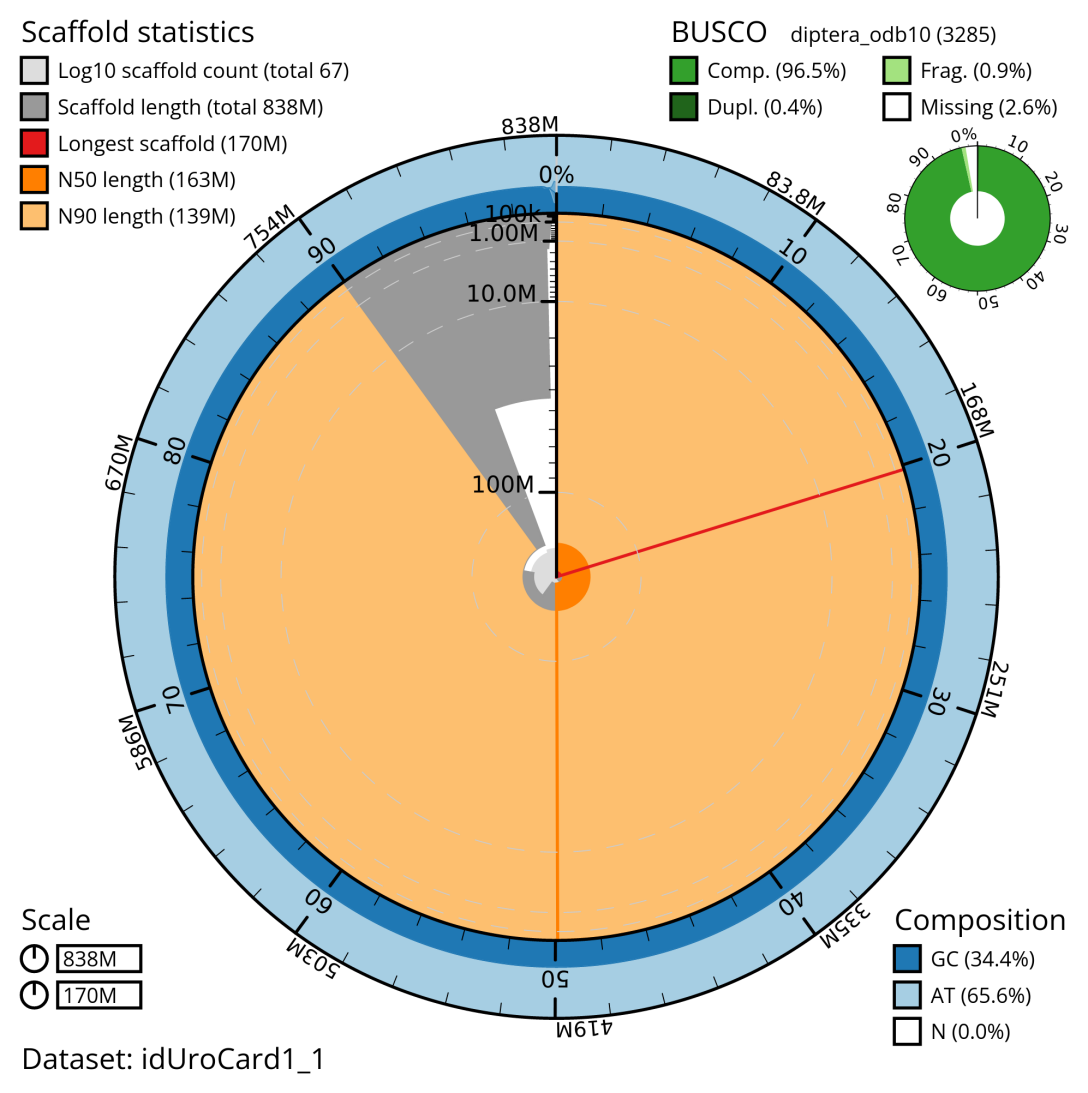
Genome assembly of *Urophora cardui*, idUroCard1.1: metrics. The BlobToolKit snail plot shows N50 metrics and BUSCO gene completeness. The main plot is divided into 1,000 size-ordered bins around the circumference with each bin representing 0.1% of the 837,836,207 bp assembly. The distribution of scaffold lengths is shown in dark grey with the plot radius scaled to the longest scaffold present in the assembly (169,694,088 bp, shown in red). . Orange and pale-orange arcs show the N50 and N90 scaffold lengths (162,960,411 and 139,447,831 bp), respectively. The pale grey spiral shows the cumulative scaffold count on a log scale with white scale lines showing successive orders of magnitude. The blue and pale-blue area around the outside of the plot shows the distribution of GC, AT and N percentages in the same bins as the inner plot. A summary of complete, fragmented, duplicated and missing BUSCO genes in the diptera_odb10 set is shown in the top right. An interactive version of this figure is available at https://blobtoolkit.genomehubs.org/view/idUroCard1_1/dataset/idUroCard1_1/snail.

**Figure 3.**
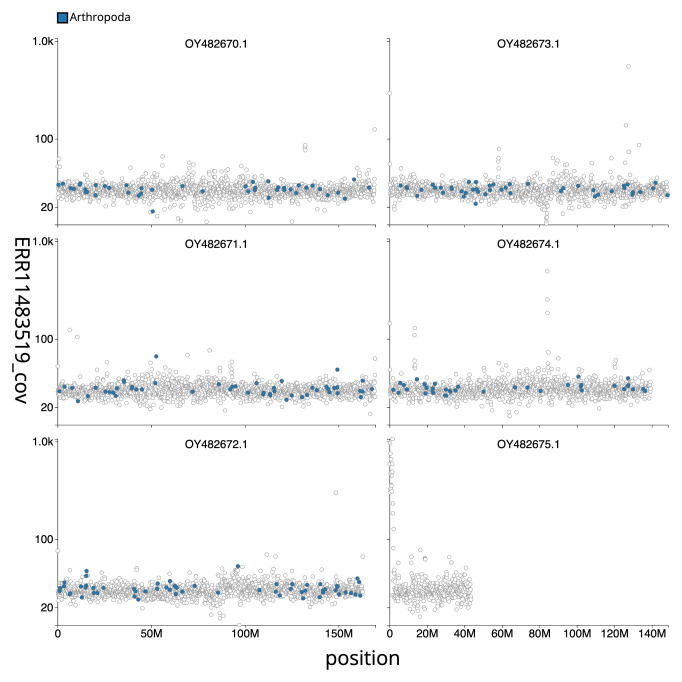
Genome assembly of *Urophora cardui*, idUroCard1.1: Distribution plot of base coverage in ERR11483519 against position for sequences in the assembly. Windows of 100 kb are coloured by phylum. The assembly has been filtered to exclude sequences with length < 2,550,000. An interactive version of this figure is available here.

**Figure 4.**
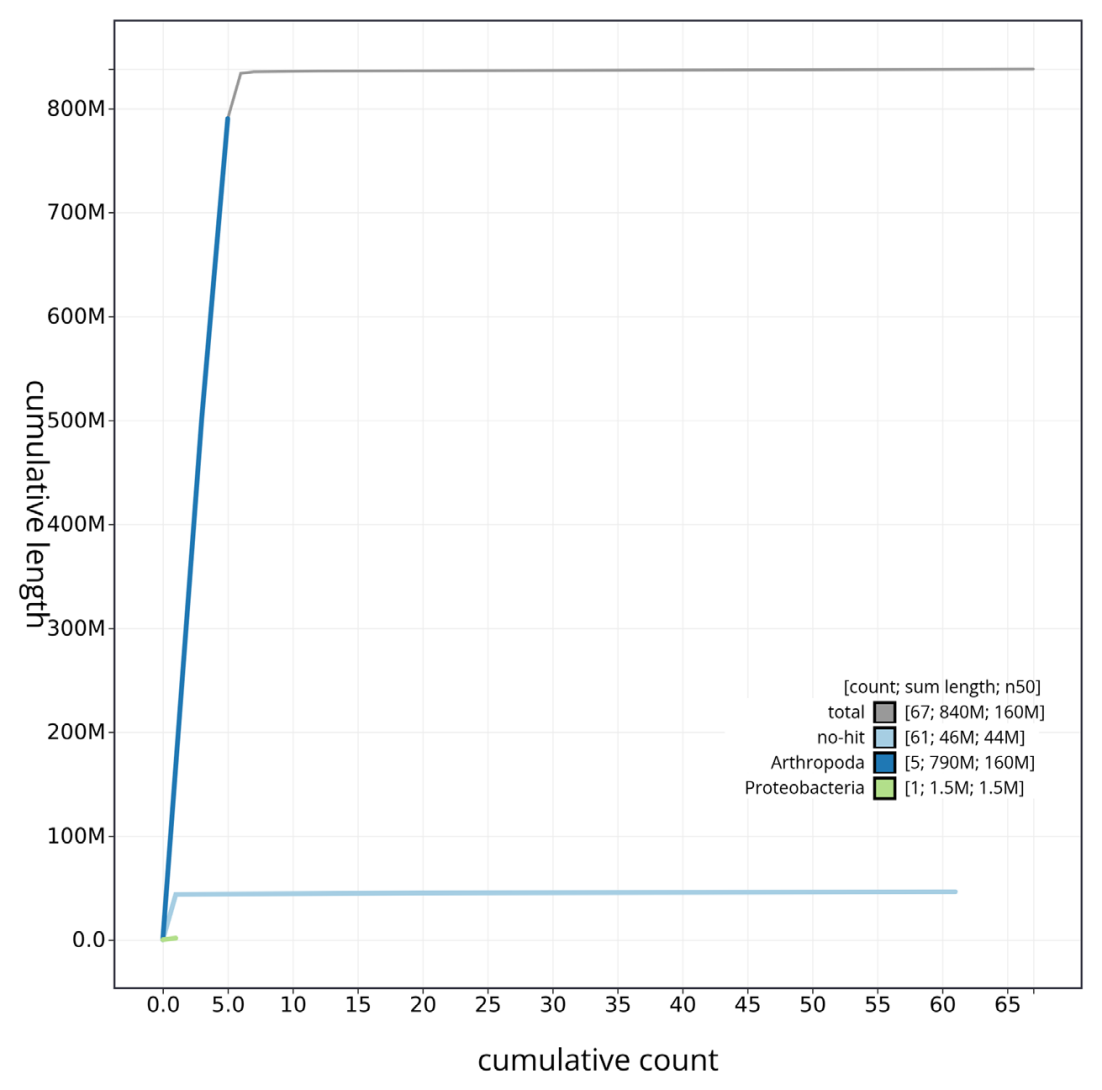
Genome assembly of *Urophora cardui* idUroCard1.1: BlobToolKit cumulative sequence plot. The grey line shows cumulative length for all sequences. Coloured lines show cumulative lengths of sequences assigned to each phylum using the buscogenes taxrule. An interactive version of this figure is available at https://blobtoolkit.genomehubs.org/view/idUroCard1_1/dataset/idUroCard1_1/cumulative.

**Figure 5.**
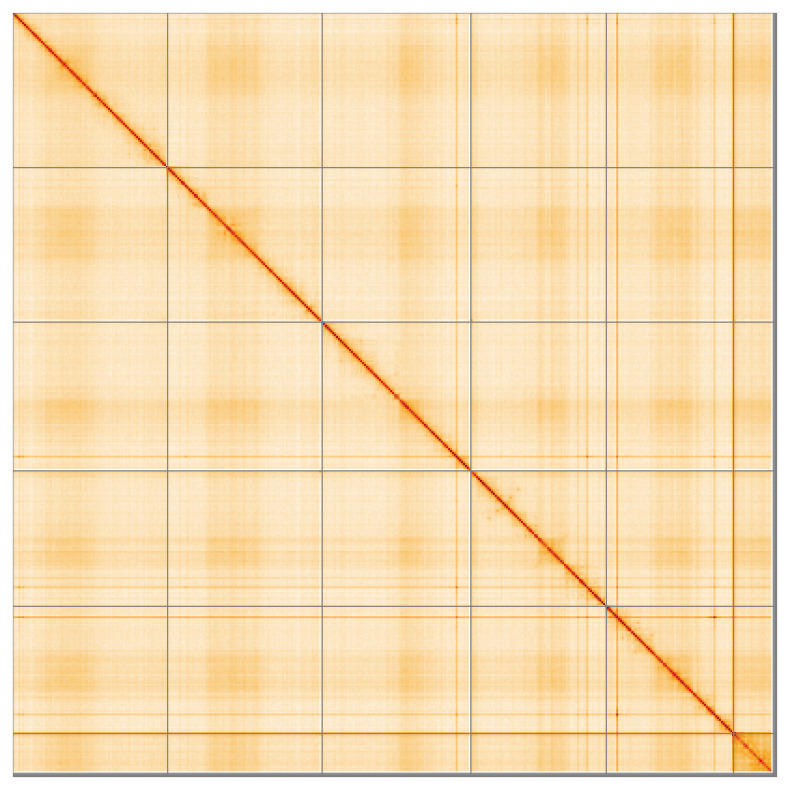
Genome assembly of *Urophora cardui* idUroCard1.1: Hi-C contact map of the idUroCard1.1 assembly, visualised using HiGlass. Chromosomes are shown in order of size from left to right and top to bottom. An interactive version of this figure may be viewed at https://genome-note-higlass.tol.sanger.ac.uk/l/?d=L-VARUgZTPCe69o4zIhIHA.

Manual assembly curation corrected 124 missing joins or mis-joins and five haplotypic duplications, reducing the scaffold number by 43.7%, and decreasing the scaffold N50 by 0.5%. The final assembly has a total length of 837.80 Mb in 66 sequence scaffolds with a scaffold N50 of 163.0 Mb ( Table 2). The total count of gaps in the scaffolds is 575. The snail plot in Figure 2 provides a summary of the assembly statistics, while Figure 3 shows the distribution of base coverage against position for sequences in each chromosome. The cumulative assembly plot in Figure 4 shows curves for subsets of scaffolds assigned to different phyla. Most (99.78%) of the assembly sequence was assigned to 6 chromosomal-level scaffolds. Chromosome-scale scaffolds confirmed by the Hi-C data are named in order of size ( Figure 5; Table 3). While not fully phased, the assembly deposited is of one haplotype. Contigs corresponding to the second haplotype have also been deposited. The mitochondrial genome was also assembled and can be found as a contig within the multifasta file of the genome submission.

**Table 2.** Genome assembly data for *Urophora cardui*, idUroCard1.1.

Genome assembly		
Assembly name	idUroCard1.1	
Assembly accession	GCA_960531455.1	
*Accession of alternate haplotype*	*GCA_960531475.1*	
Span (Mb)	837.80	
Number of contigs	642	
Contig N50 length (Mb)	3.0	
Number of scaffolds	66	
Scaffold N50 length (Mb)	163.0	
Longest scaffold (Mb)	171.21	
Assembly metrics *		*Benchmark*
Consensus quality (QV)	60.8	*≥ 50*
*k*-mer completeness	100.0%	*≥ 95%*
BUSCO **	C:96.5%[S:96.1%,D:0.4%],F:0.9%,M:2.6%,n:3,285	*C ≥ 95%*
Percentage of assembly mapped to chromosomes	99.78%	*≥ 95%*
Sex chromosomes	Not identified	*localised homologous pairs*
Organelles	Mitochondrial genome: 20.37 kb	*complete single alleles*

* Assembly metric benchmarks are adapted from column VGP-2020 of “Table 1: Proposed standards and metrics for defining genome assembly quality” from Rhie *et al.* (2021). ** BUSCO scores based on the diptera_odb10 BUSCO set using version 5.3.2. C = complete [S = single copy, D = duplicated], F = fragmented, M = missing, n = number of orthologues in comparison. A full set of BUSCO scores is available at https://blobtoolkit.genomehubs.org/view/idUroCard1_1/dataset/idUroCard1_1/busco.

**Table 3.** Chromosomal pseudomolecules in the genome assembly of *Urophora cardui*, idUroCard1.

INSDC accession	Name	Length (Mb)	GC%
OY482670.1	1	169.38	34.5
OY482671.1	2	169.69	34.5
OY482672.1	3	162.96	34.5
OY482673.1	4	148.57	34.5
OY482674.1	5	139.45	34.5
OY482675.1	6	43.56	34.5
OY482676.1	MT	0.02	17.0

The estimated Quality Value (QV) of the final assembly is 60.8 with *k*-mer completeness of 100.0%, and the assembly has a BUSCO v5.3.2 completeness of 96.5% (single = 96.1%, duplicated = 0.4%), using the diptera_odb10 reference set ( *n* = 3,285).

Metadata for specimens, BOLD barcode results, spectra estimates, sequencing runs, contaminants and pre-curation assembly statistics are given at https://links.tol.sanger.ac.uk/species/503482.

## Methods

### Sample acquisition

An adult female *Urophora cardui* (specimen ID Ox002373, ToLID idUroCard1) was netted in Wytham Woods, Oxfordshire, UK (latitude 51.77, longitude –1.34) on 2022-05-28. The specimen was collected by Leila Franzen and Liam Crowley (University of Oxford), identified by Liam Crowley (University of Oxford) and preserved on dry ice.

The initial species identification was verified by an additional DNA barcoding process according to the framework developed by Twyford *et al*. (2024). A small sample was dissected from the specimens and stored in ethanol, while the remaining parts of the specimen were shipped on dry ice to the Wellcome Sanger Institute (WSI). The tissue was lysed, the COI marker region was amplified by PCR, and amplicons were sequenced and compared to the BOLD database, confirming the species identification ( Crowley *et al.*, 2023). Following whole genome sequence generation, the relevant DNA barcode region was also used alongside the initial barcoding data for sample tracking at the WSI ( Twyford *et al.*, 2024). The standard operating procedures for Darwin Tree of Life barcoding have been deposited on protocols.io ( Beasley *et al.*, 2023).

### Nucleic acid extraction

The workflow for high molecular weight (HMW) DNA extraction at the Wellcome Sanger Institute (WSI) Tree of Life Core Laboratory includes a sequence of core procedures: sample preparation and homogenisation, DNA extraction, fragmentation and purification. Detailed protocols developed by the WSI Tree of Life laboratory are publicly available on protocols.io ( Denton *et al.*, 2023b).

In sample preparation, the idUroCard1 sample was weighed and dissected on dry ice ( Jay *et al.*, 2023). Tissue from the whole organism was homogenised using a PowerMasher II tissue disruptor ( Denton *et al.*, 2023a). HMW DNA was extracted using the Automated MagAttract v1 protocol ( Sheerin *et al.*, 2023). DNA was sheared into an average fragment size of 12–20 kb in a Megaruptor 3 system with speed setting 30 ( Todorovic *et al.*, 2023). Sheared DNA was purified by solid-phase reversible immobilisation, using AMPure PB beads to eliminate shorter fragments and concentrate the DNA ( Strickland *et al.*, 2023). The concentration of the sheared and purified DNA was assessed using a Nanodrop spectrophotometer and Qubit Fluorometer using the Qubit dsDNA High Sensitivity Assay kit. Fragment size distribution was evaluated by running the sample on the FemtoPulse system.

### Sequencing

Pacific Biosciences HiFi circular consensus DNA sequencing libraries were constructed according to the manufacturers’ instructions. DNA sequencing was performed by the Scientific Operations core at the WSI on a Pacific Biosciences Sequel IIe instrument. Hi-C data were also generated from whole organism tissue of idUroCard1 using the Arima-HiC v2 kit. The Hi-C sequencing was performed using paired-end sequencing with a read length of 150 bp on the Illumina NovaSeq 6000 instrument.

### Genome assembly, curation and evaluation

***Assembly***

The HiFi reads were first assembled using Hifiasm ( Cheng *et al.*, 2021) with the --primary option. Haplotypic duplications were identified and removed using purge_dups ( Guan *et al.*, 2020). The Hi-C reads were mapped to the primary contigs using bwa-mem2 ( Vasimuddin *et al.*, 2019). The contigs were further scaffolded using the provided Hi-C data ( Rao *et al.*, 2014) in YaHS ( Zhou *et al.*, 2023) using the --break option. The scaffolded assemblies were evaluated using Gfastats ( Formenti *et al.*, 2022), BUSCO ( Manni *et al.*, 2021) and MERQURY.FK ( Rhie *et al.*, 2020).

The mitochondrial genome was assembled using MitoHiFi ( Uliano-Silva *et al.*, 2023), which runs MitoFinder ( Allio *et al.*, 2020) and uses these annotations to select the final mitochondrial contig and to ensure the general quality of the sequence.

***Assembly curation***

The assembly was decontaminated using the Assembly Screen for Cobionts and Contaminants (ASCC) pipeline (article in preparation). Flat files and maps used in curation were generated in TreeVal ( Pointon *et al.*, 2023). Manual curation was primarily conducted using PretextView ( Harry, 2022), with additional insights provided by JBrowse2 ( Diesh *et al.*, 2023) and HiGlass ( Kerpedjiev *et al.*, 2018). Scaffolds were visually inspected and corrected as described by Howe *et al*. (2021). Any identified contamination, missed joins, and mis-joins were corrected, and duplicate sequences were tagged and removed. The curation process is documented at https://gitlab.com/wtsi-grit/rapid-curation (article in preparation).

***Evaluation of the final assembly***

A Hi-C map for the final assembly was produced using bwa-mem2 ( Vasimuddin *et al.*, 2019) in the Cooler file format ( Abdennur & Mirny, 2020). To assess the assembly metrics, the *k*-mer completeness and QV consensus quality values were calculated in Merqury ( Rhie *et al.*, 2020). This work was done using the “sanger-tol/readmapping” ( Surana *et al.*, 2023a) and “sanger-tol/genomenote” ( Surana *et al.*, 2023b) pipelines. The genome readmapping pipelines were developed using the nf-core tooling ( Ewels *et al.*, 2020), use MultiQC ( Ewels *et al.*, 2016), and make extensive use of the Conda package manager, the Bioconda initiative ( Grüning *et al.*, 2018), the Biocontainers infrastructure ( da Veiga Leprevost *et al.*, 2017), and the Docker ( Merkel, 2014) and Singularity ( Kurtzer *et al.*, 2017) containerisation solutions. The genome was also analysed within the BlobToolKit environment ( Challis *et al.*, 2020) and BUSCO scores ( Manni *et al.*, 2021; Simão *et al.*, 2015) were calculated.

Table 4 contains a list of relevant software tool versions and sources.

**Table 4.** Software tools: versions and sources.

Software tool	Version	Source
BlobToolKit	4.2.1	https://github.com/blobtoolkit/blobtoolkit
BUSCO	5.3.2	https://gitlab.com/ezlab/busco
bwa-mem2	2.2.1	https://github.com/bwa-mem2/bwa-mem2
Gfastats	1.3.6	https://github.com/vgl-hub/gfastats
Hifiasm	0.16.1-r375	https://github.com/chhylp123/hifiasm
HiGlass	1.11.6	https://github.com/higlass/higlass
Merqury.FK	d00d98157618f4e8d1a9190026b19 b471055b22e	https://github.com/thegenemyers/MERQURY.FK
MitoHiFi	2	https://github.com/marcelauliano/MitoHiFi
PretextView	0.2	https://github.com/wtsi-hpag/PretextView
purge_dups	1.2.3	https://github.com/dfguan/purge_dups
sanger-tol/genomenote	v1.0	https://github.com/sanger-tol/genomenote
sanger-tol/readmapping	1.1.0	https://github.com/sanger-tol/readmapping/tree/1.1.0
YaHS	yahs-1.1.91eebc2	https://github.com/c-zhou/yahs

### Wellcome Sanger Institute – Legal and Governance

The materials that have contributed to this genome note have been supplied by a Darwin Tree of Life Partner. The submission of materials by a Darwin Tree of Life Partner is subject to the **‘Darwin Tree of Life Project Sampling Code of Practice’**, which can be found in full on the Darwin Tree of Life website here. By agreeing with and signing up to the Sampling Code of Practice, the Darwin Tree of Life Partner agrees they will meet the legal and ethical requirements and standards set out within this document in respect of all samples acquired for, and supplied to, the Darwin Tree of Life Project.

Further, the Wellcome Sanger Institute employs a process whereby due diligence is carried out proportionate to the nature of the materials themselves, and the circumstances under which they have been/are to be collected and provided for use. The purpose of this is to address and mitigate any potential legal and/or ethical implications of receipt and use of the materials as part of the research project, and to ensure that in doing so we align with best practice wherever possible. The overarching areas of consideration are:

•   Ethical review of provenance and sourcing of the material

•   Legality of collection, transfer and use (national and international) 

Each transfer of samples is further undertaken according to a Research Collaboration Agreement or Material Transfer Agreement entered into by the Darwin Tree of Life Partner, Genome Research Limited (operating as the Wellcome Sanger Institute), and in some circumstances other Darwin Tree of Life collaborators.

## Data Availability

European Nucleotide Archive: Urophora cardui (thistle gall fly). Accession number PRJEB62614; https://identifiers.org/ena.embl/PRJEB62614 ( Wellcome Sanger Institute, 2024). The genome sequence is released openly for reuse. The *Urophora cardui* genome sequencing initiative is part of the Darwin Tree of Life (DToL) project. All raw sequence data and the assembly have been deposited in INSDC databases. The genome will be annotated using available RNA-Seq data and presented through the Ensembl pipeline at the European Bioinformatics Institute. Raw data and assembly accession identifiers are reported in Table 1 and Table 2.

## Author information

Members of the University of Oxford and Wytham Woods Genome Acquisition Lab are listed here: https://doi.org/10.5281/zenodo.12157525.

Members of the Darwin Tree of Life Barcoding collective are listed here: https://doi.org/10.5281/zenodo.12158331

Members of the Wellcome Sanger Institute Tree of Life Management, Samples and Laboratory team are listed here: https://doi.org/10.5281/zenodo.12162482.

Members of Wellcome Sanger Institute Scientific Operations: Sequencing Operations are listed here: https://doi.org/10.5281/zenodo.12165051.

Members of the Wellcome Sanger Institute Tree of Life Core Informatics team are listed here: https://doi.org/10.5281/zenodo.12160324.

Members of the Tree of Life Core Informatics collective are listed here: https://doi.org/10.5281/zenodo.12205391.

Members of the Darwin Tree of Life Consortium are listed here: https://doi.org/10.5281/zenodo.4783558.
