# Supplementary material for: The genome sequence of the thistle gall fly, Urophora cardui (Linnaeus 1758)
Source: Wellcome Open Res. 2024 Sep 3;9:502. [Version 1] doi: 10.12688/wellcomeopenres.22919.1 (PMC11427867; doi:10.12688/wellcomeopenres.22919.1)
Supplement: Peer review history

# Reviewer report: Reviewer response for version 1

**DOI:** 10.21956/wellcomeopenres.25236.r100659

**Published:** 2024-09-27

**Authors:** Craig Wilding

**Affiliations:** 1 Liverpool John Moores University, Liverpool, UK

Franzen et al. present a high-quality genome sequence of the fly *Urophora cardui*. The introduction is clear and presents useful information on the biology, distribution, and use of this species in biological control.

 The methods are clear. The sequence is extremely high quality - chromosomal length (6 chromosomal pseudomolecules) and with a very high BUSCO completeness.

**My only comments are:**

- Figure 1 - I expect the tube is used for scale, but without knowledge of the size of the tube this is not particularly useful - can a scale bar be added or the legend state the length of the tube?
- Figure 3 - I am not clear exactly what this is showing. The blue dots are Arthropoda. Does this mean these are example Arthropoda to demonstrate that the sequences from *Urophora cardui* are of a similar value or the average value of other arthropods (which does not really make sense in this plot)? If the blue dots are *Urophora cardui* (which is an arthropod) what are the other dots? This plot just needs a better explanation for those unfamiliar with such a presentation. I am also wondering why there is a peak in coverage at the start of pseudomolecule OY482675.1? This is not mentioned in the text.

Are sufficient details of methods and materials provided to allow replication by others?

Yes

Is the rationale for creating the dataset(s) clearly described?

Yes

Are the datasets clearly presented in a useable and accessible format?

Yes

Are the protocols appropriate and is the work technically sound?

Yes

Reviewer Expertise:

Genetics/genomics of mosquitoes, spiders and sea anemones

I confirm that I have read this submission and believe that I have an appropriate level of expertise to confirm that it is of an acceptable scientific standard.

# Reviewer report: Reviewer response for version 1

**DOI:** 10.21956/wellcomeopenres.25236.r99474

**Published:** 2024-09-27

**Authors:** Daniel Gebert

**Affiliations:** 1 University of Cambridge, England, UK

The authors describe a genome assembly from an individual female thistle gall fly ( *Urophora cardui*), which they generated using PacBio sequencing, followed by HiC scaffolding of the initially assembled contigs into chromosomes (chromosomal pseudomolecules), including the mitochondrial genome. A total of 99.78% of the assembly sequence (altogether 837.80 Mb) was assigned to six chromosome-level scaffolds, named in order of their size. However, sex chromosomes (likely the X chromosome in this female specimen) were not identified, which is unsurprising since, in close relatives such as *Bactrocera dorsalis*, which also has six chromosomes, the sex chromosomes remain unidentified as well.

 The methodology is sound, and the assembly evaluation is rigorous. While the sequence report section itself is very short and could benefit from further elaboration, it contains the most important basic information for a typical DToL assembly report. The authors also provide a detailed description of the significance of this species and the value of having a high-quality genome assembly, which is very convincing.

 However, it would be beneficial to state in more detail which options were used for specific purposes during assembly and scaffolding. For instance, what is the purpose of the Hifiasm --primary option, and why was it chosen? Similarly, what is the --break option in yahs, and how does it affect scaffolding? What was the mapq cutoff? Including these details would help the reader fully understand and recapitulate the methodology.

Are sufficient details of methods and materials provided to allow replication by others?

Partly

Is the rationale for creating the dataset(s) clearly described?

Yes

Are the datasets clearly presented in a useable and accessible format?

Yes

Are the protocols appropriate and is the work technically sound?

Yes

Reviewer Expertise:

Genome Biology, Evolution, Computational Biology

I confirm that I have read this submission and believe that I have an appropriate level of expertise to confirm that it is of an acceptable scientific standard.

# Reviewer report: Reviewer response for version 1

**DOI:** 10.21956/wellcomeopenres.25236.r100623

**Published:** 2024-09-26

**Authors:** Jason Charamis

**Affiliations:** 1 Foundation for Research and Technology - Hellas, Irákleion, Greece

This work describes the genome sequence of the Canada Thistle Gall Fly. The work is technically sound, both in terms of wet lab and computational approaches. The methods and resulting assembly well described in the article and will be an important contribution to ecological genomic studies. I suggest  indexing this paper.

Are sufficient details of methods and materials provided to allow replication by others?

Yes

Is the rationale for creating the dataset(s) clearly described?

Yes

Are the datasets clearly presented in a useable and accessible format?

Yes

Are the protocols appropriate and is the work technically sound?

Yes

Reviewer Expertise:

Arthropod Comparative and Computational Genomics

I confirm that I have read this submission and believe that I have an appropriate level of expertise to confirm that it is of an acceptable scientific standard.

# Reviewer report: Reviewer response for version 1

**DOI:** 10.21956/wellcomeopenres.25236.r100629

**Published:** 2024-09-25

**Authors:** Henrique Antoniolli

**Affiliations:** 1 Universidade Federal do Rio Grande do Sul (UFRGS), Porto Alegre, Brazil

The present study reports a highly contiguous genome assembly of *Urophora cardui*, an important fruit fly used as biological control against an invasive weed in North America. The authors bring a clear and concise introduction, providing valuable information regarding its general biology and ecology; in this sense, the rationale for creating these datasets is clearly stated throughout the text: gall evolution, biogeography, insect-plant interactions, and population genomics. Protocols are well described and provided in the methodology section as supplementary material, fully accessible in other repositories. Accession numbers for both raw and assembled data are given, enabling replication by others. Main statistics are also provided, showing that the assembly reached satisfactory levels of completeness. Although annotation of the nuclear genome was not performed, authors stated that this will be provided soon by employing RNA-seq data; annotation of the mitochondrial genome should be performed as well. The paper also lack an estimation of the repetitive content, which may be performed in future studies.

Are sufficient details of methods and materials provided to allow replication by others?

Yes

Is the rationale for creating the dataset(s) clearly described?

Yes

Are the datasets clearly presented in a useable and accessible format?

Yes

Are the protocols appropriate and is the work technically sound?

Yes

Reviewer Expertise:

Genomics; transposable elements

I confirm that I have read this submission and believe that I have an appropriate level of expertise to confirm that it is of an acceptable scientific standard.
